# Development of a remineralizing calcium phosphate nanoparticle-containing self-etching system for orthodontic bonding

**DOI:** 10.1007/s00784-022-04767-5

**Published:** 2022-11-02

**Authors:** Noor M. H. Garma, Ali I. Ibrahim

**Affiliations:** 1 Orthodontic Department, College of Dentistry, University of Baghdad, Bab Al-Moadham Campus, Floor 4, Baghdad, Iraq; 2 Centre for Oral, Clinical and Translational Sciences, Faculty of Dentistry, Oral & Craniofacial Sciences, King’s College London, London, UK

**Keywords:** Enamel remineralization, Self-etch primers, Bond Strength, Calcium phosphates, Enamel damage, Adhesive residue

## Abstract

**Objectives:**

This study aimed to incorporate hydroxyapatite nanoparticles (nHA) or amorphous calcium phosphate nanoparticles (nACP) into a self-etch primer (SEP) to develop a simplified orthodontic bonding system with remineralizing and enamel preserving properties.

**Materials and Methods:**

nHA and nACP were incorporated into a commercial SEP (Transbond™ plus) in 7% weight ratio and compared with the plain SEP as a control. Shear bond strengths (SBS), enamel damage, and adhesive remnant index (ARI) scores were evaluated at 24 h and post 5000 thermocycling. Field-emission scanning electron microscope (FESEM) was used to inspect the distribution of the nanoparticles in the experimental SEPs and evaluate the enamel surface integrity both before bracket bonding and post bracket debonding. Phase determination and remineralizing capability of the modified SEP were characterized by X-ray diffraction and Raman spectroscopy, respectively.

**Results:**

The addition of nHA or nACP to the SEP significantly reduced the SBS, ARI, and enamel damage (*p* < 0.05) as compared to the control SEP; however, only nHA-SEP survived the thermocycling protocol and yielded acceptable SBS (13.38 MPa). Enamel remineralizing ability of the developed nHA-SEP was confirmed by both FESEM images and Raman phosphate map.

**Conclusions:**

Incorporating nHA into SEP resulted in clinically acceptable bond strengths with remineralizing ability.

**Clinical relevance:**

The newly developed nHA-SEP has unprecedented ability to simultaneously etch, prime, and remineralize the enamel in a single step leaving immaculate enamel surface with the potential of saving cost and time at the post-debonding step.

## Introduction

Orthodontic treatment is commonly used to correct different malocclusions and improve facial aesthetics [1]. However, multibracket orthodontic treatment is frequently accompanied by undesirable side effects, and the most important is development of white spot lesions [2, 3]. The second most challenging problem is enamel damage that ensues during bracket bonding/debonding procedures, as enamel tissue loss occurs during polishing and etching steps, and further enamel damage in the form of cracks or fractures can occur during bracket debonding [4–6]. Moreover, enamel scratching is encountered during adhesive remnant (AR) removal using various cleaning techniques at the postbracket debonding step [7].

Conventional enamel etching (CAE) with phosphoric acid results in a roughened, porous layer that provides a long-lasting bond to enamel, which is desirable for preventing premature loss of brackets [8]. However, as the bonding forces increase, so does the frequency of enamel cracks and bracket fracture during bracket debonding [4, 9]. Therefore, enamel damage is commonly seen when debonding metallic brackets at the completion of treatment [10, 11] and is even more evident when debonding ceramic brackets with higher bond strengths [4]. Accordingly, effort is ongoing to develop techniques constituting alternatives to the CAE method in an attempt to eliminate these iatrogenic effects. Examples include the use of a laser, resin-modified glass ionomer cements (RMGIC), and self-etching primers for orthodontic bonding. Enamel etching with a laser can produce acceptable bond strengths but is associated with a fractured, grooved enamel surfaces and abundant AR post bracket debonding comparable to that of the CAE approach [5], whereas RMGIC leaves minimal AR and enamel damage but is associated with inadequate bracket retention [12]. On the other hand, self-etching primers (SEPs) provide simultaneous etching and priming functions, which is more convenient for both the clinician and patient due to chair-side time savings and reliable bond strengths [13, 14]. However, there is debate as to whether the amount of adhesive left after debonding is less than or the same as that of the CAE technique, and enamel damage after bracket debonding could not be eliminated [4, 15, 16].

Biomimetic enamel regeneration with calcium-phosphate nanoparticles (nCaP) is considered a promising developmental therapy for enamel repair and remineralization [17, 18]. A recent study attempted to harness the advantages of nCaP in orthodontics through direct topical application around orthodontic brackets to combat enamel demineralization [19]. However, the topical application was premised on patient compliance, which is unpredictable and unreliable. In addition, nCaPs were incorporated into composite adhesives [20, 21] or combined with other antimicrobial agents in an orthodontic bonding material [22] depending on the ion-release concept, which is known for its short-term release effectiveness. Moreover, the reductions seen in the amount of AR and/or enamel damage occurring at bracket debonding were not significant in these attempts [23, 24].

Research is lacking on development of an orthodontic bonding system that combines the simplified technique of SEP and the remineralizing effect of nCaP. Therefore, this study is designed to develop a novel orthodontic self-etching system that is capable of producing clinically acceptable bond strengths, inducing enamel remineralization, decreasing enamel damage at bracket removal, and leaving minimal AR on enamel after debonding. The study rationale was based on inducing changes in the enamel surface chemistry to enhance its resistance to demineralization through incorporation of nHA and nACP into a SEP monomer. Herein, nHA and nACP were utilized due to their self-assembly properties and structures that are chemically analogous to native enamel [18]. The following hypotheses were tested: (1) A new orthodontic nCaP-SEP bonding system can be developed without compromising the bracket bond strength. (2) The new nCaP-SEP system would decrease the detrimental effects of SEP alone on the enamel surface both at and after bracket debonding.

## Materials and methods

### Teeth collection and preparation

A total of 139 human premolar and molar teeth (extracted for orthodontic purposes) were collected from 12- to 25-year-old patients after obtaining ethical approval from the Ethics Committee at the College of Dentistry/ University of Baghdad (Ref. number: 208). The criteria for tooth selection included intact buccal enamel surface without previous treatments with chemical agents (e.g., etchants, hydrogen peroxide), no detectable caries, enamel cracks, or irregularities examined at × 10 magnification using a stereomicroscope. After extraction, the teeth were cleaned with a scalpel under running water, stored in a 1% chloramine-T trihydrate solution for 1 week, and then stored in distilled water until the time of bonding (ISO/TS 11,405:2015, testing of adhesion to tooth structure) [25].

**Fig. 1:**
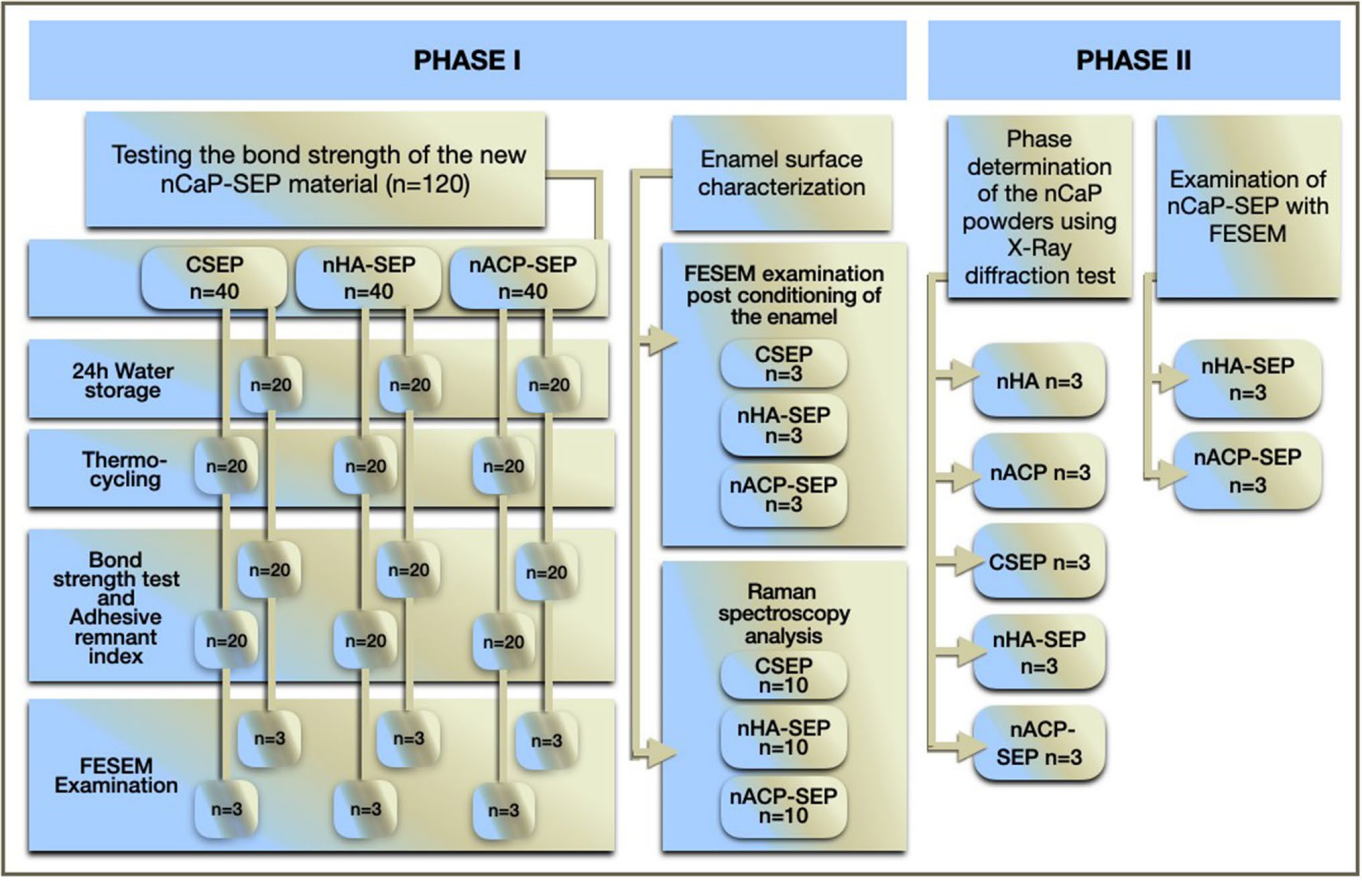
A flowchart summarizing the experiments implemented in the study

The study involved two phases; the implemented experiments of the two phases are illustrated in Fig. 1.

#### Phase I: development, testing, and characterization of nCaP-SEP

##### Development of nCaP-SEP formulations

Commercially available hydroxyapatite nanoparticles (nHA) [Ca_5_(OH)(PO_4_)_3_] (Sigma-Aldrich, USA; particle size < 200 nm) and amorphous calcium phosphate nanoparticles (nACP) [Ca_2_O_7_P_2*_H_2_O] (Sigma-Aldrich, USA, particle size < 150 nm) were used to formulate the experimental nHA-SEP and nACP-SEP, respectively. A concentration of 7% wt from each nCaP compound was prepared in a weight:weight ratio and added to the plain SEP; the exact weight of the nCaP and SEP was measured using a sensitive weight balance (ALJ_N/ALS_N, KERN & SOHN GmbH, Germany). The nano-powder was added to an Eppendorf tube containing the plain photo-polymerizable single-step SEP primer (Transbond™ plus, 3 M Unitek, USA), its compositions are enclosed in two bubble reservoirs: the first contains methacrylated phosphoric acid esters, bis-GMA, camphor quinone, and stabilizers; the second one contains water, HEMA, polyalkene acid, complex fluoride, and stabilizers. Each Eppendorf tube was shaken at constant agitation for 20 s using an electric shaker until a homogenous suspension of the assigned nCaP-SEP was obtained. The formulations were freshly and individually prepared before use in each subsequent bonding procedure. The preparation of the new nCaP-SEP was conducted in a UV light-protected room under ambient conditions (20–23 °C and 50–60% humidity). The plain single-step SEP primer was used as a control (CSEP). The pH values of the control and experimental formulations were measured using a digital pH meter with a flat-end electrode (S450CD, Sensorex, USA).

##### Bonding procedure

One hundred twenty premolar teeth were mounted vertically in acrylic blocks using a surveyor to align the buccal surface of each tooth so that the force runs parallel to the bonded bracket base. The buccal surface was polished with a fluoride-free pumice slurry and rubber cups for 10 s, followed by thorough water rinsing (10 s) and oil-free air dryness (10 s). The teeth of all tested groups were then bonded according to the manufacturer’s recommended protocol for using the plain SEP. The SEP material was applied onto the buccal enamel surface with continuous rubbing for 5 s, then dried lightly with compressed air for 2 s. Metallic (stainless steel) upper premolar brackets (Pinnacle, Orthotechnology, USA) were used for bonding. The bracket base was loaded with a thin layer of the composite adhesive (Transbond XT, 3 M Unitek, USA) and placed onto the buccal surface with a 300 g pressing force for 3 s measured using a force gauge, then exposed to a light-emitting diode curing light (Elipar™ DeepCure-L, 3 M ESPE, Germany) for a total of 20 s (10 s from each of the mesial and distal side) [11, 15].

##### Testing the new nCaP-SEP formulation after water storage and thermocycling

The mounted premolar teeth were randomly assigned into three groups (*n* = 40) according to the SEP formulation used for brackets bonding:

- Teeth bonded with the plain SEP referred to as CSEP (control group).
- Teeth bonded with the primer of 7% nHA (nHA-SEP).
- Teeth bonded with the primer of 7% nACP (nACP-SEP).

All bonded teeth were stored in distilled water for 24 h at 37 °C. Then, each group was further subdivided into two subgroups (*n* = 20) according to the timing of SBS testing: one subgroup was debonded after 24 h water storage, and the other was further thermocycled between cold and hot water baths of 5 and 55 °C for 5000 cycles with a dwelling time of 30 s and transfer time between baths of 5 s [11].

##### SBS, ARI, and enamel damage evaluation

To measure the SBS, a chisel on a universal testing machine (WDW-50E, laryee, China) was applied to the upper part of the bracket base, parallel to the bonded interface. An occluso-gingival force was loaded at a cross-head speed of 0.5 mm/min until bracket failure [11, 21]. The SBS was calculated in MPa by dividing the load at bracket detachment (recorded in Newton) by the bracket base surface area. After brackets debonding, all the specimens were coded and examined by an experienced clinician who was blinded to which formulation of SEP was used during bracket bonding. Then, each tooth surface was observed under a stereomicroscope (Biovision BVS 3200 Hamilton, Altay, Germany) at × 10 magnification to examine enamel damage (cracks/fracture) and the failure mode or ARI. The ARI is based on the amount of remaining adhesive material on enamel surface using the following scoring system: 0, no adhesive left on enamel surface; 1, less than half of adhesive left; 2, more than half of adhesive left; and 3, all adhesive left with a distinct imprint of the bracket mesh [26]. Three randomly selected specimens of each subgroup were sputter-coated with gold nanoparticles (30 nm) and examined using field-emission scanning electron microscope machine (FESEM) (Nova NanoSEM 450, FEI, Holland) at an accelerating voltage of 10 kV under low-vacuum operations.

##### X-ray diffraction test

Phase determination of the nCaP powders, CSEP, nHA-SEP, and nACP-SEP was performed using XRD. Three samples of each of the control and aforementioned experimental SEP types were prepared to minimize the variability of specimens’ homogeneity [27] using custom-made teflon mold. Each disc (5 mm diameter × 2 mm thickness) was prepared by direct dispensing of the liquid primer until filling the mold. After curing of the discs, they were kept for 24 h in an open glass container to obtain a solid dry mass before grinding into fine powder by hand-milling using an agate mortar and pestle. The powder samples were examined using a diffractometer (LabX-XRD-6000, Shimadzu, Japan) in a flat-plate geometry using continuous scan mode with a range of 5.0–60.0°, step size 0.05°, Cu target, 40 kV, and 30 mA X-ray radiation.

##### Examination of nCaP-SEP with FESEM

Six discs were prepared using the same procedure mentioned in the XRD test, three discs for polymerized nHA-SEP, and three for polymerized nACP-SEP. Each disc was divided into two halves, fixed with a conductive carbon tape such that the inner side of the cleavage is directed outward, then examined using FESEM.

#### Phase II: Enamel surface characterization

##### FESEM examination post conditioning of the enamel

To evaluate the etching effect induced by the experimental nCaP-SEP in comparison with the CSEP, three teeth were used to represent each group. The buccal enamel surfaces were conditioned with the assigned SEP for 5 s, rinsed under running water for 30 s, and dried with oil-free airstream [28]. The crown of each tooth was sectioned mesio-distally through the occlusal central fossae using a metal abrasive disk under running water to obtain the buccal halves. All samples were kept dry at ambient laboratory conditions for 24 h, then sputter-coated with gold nanoparticles (30 nm) and examined using FESEM machine at an accelerating voltage of 10 kV under low-vacuum operations. The quality of enamel etch-pattern produced was evaluated and scored according to the etch-pattern scale [29]:

- Type (1): Ideal etch, preferential dissolution of the prism cores resulting in a honeycomb-like appearance.
- Type (2): Ideal etch, preferential dissolution of the prism peripheries resulting in a reverse honeycomb or cobblestone-like appearance.
- Type (3): A mixture of type 1 and 2 patterns.
- Type (4): Pitted, roughened enamel surface. Structures look like unfinished maps or networks, enamel prisms not evident.
- Type (5): Flat smooth surface, no apparent etch.

##### Assessment of mineralization using RS analysis

Ten molar teeth were used to obtain flat enamel surface samples. Each molar tooth was cut mesio-distally through the occlusal central fossae using metal abrasive disks under running water. Then, each buccal half was mounted using cold cure acrylic and teflon mold. The outer enamel layer was removed according to a sequential polishing protocol: 600-grit for 10 s, 1200-grit for 20 s, 2500-grit for 30 s, and 4000-grit for 60 s using a water-cooled rotating polishing machine (MoPao 160E Grinder-Polisher, Shandong, China). This polishing protocol removes about 400 μm from the superficial enamel layer and gives a smooth, flat, and highly polished enamel surface [11, 30]. Then each enamel surface was divided into three zones using adhesive tapes: one unconditioned enamel, the other two treated with the plain SEP (CSEP) and nHA-SEP according to the aforementioned etching method used in FESEM analysis. The samples were kept to dry under ambient conditions for 24 h before RS examination.

The conditioned surfaces were scanned using a Raman microscope (Bruker, Senterra II, Germany) running in a grid scan mode. For each zone (untreated and conditioned zones), a Raman map was acquired at the central part covering an area of 200 × 300 μm^2^ with a resolution less than 5 μm using a 785-nm solid-state diode laser (100 mW laser power) focused using a 20 × /0.45 numerical aperture air objective. The signal was acquired using a 400 lines/mm diffraction grating centered at 750 cm^−1^ and a CCD (charge-coupled device) with an exposure time of 2 s. The microscope laser wavelength was auto-calibrated using an internal Helium/Neon ions laser with a characteristic wavelength of 632.8 nm. Raman maps were transferred into a curve-fitting software (Origin, 2018) to fit the spectra and procure the intensity mean of four phosphate (PO_4_^3−^) peaks [11, 19]. Mineralization assessment was based on comparing the alteration in the intensity mean of each PO_4_^3−^ peak in the three zones [11, 31].

### Statistical methods

The sample size was calculated using G-power version 3.1.7 (Franz Faul, Uni Kiel, Germany) according to one-way analysis of variance (ANOVA) comparing the SBS of different groups in a preliminary range-finding experiment. A sample of 18 specimens for each subgroup was needed to detect a significant difference with large effect size and 90% power 2-tailed test at 5% level of significance. Statistical analyses were conducted using the SPSS software package (version 23, SPSS Inc., Chicago, USA). Data were screened for normal distribution and homogeneity using Shapiro–Wilk and Levene’s variance tests, respectively. One-way ANOVA and Tukey HSD post hoc multi-comparison tests were performed for parametric data (SBS, Raman peak intensity) with normal distribution and equal variances. Kruskal–Wallis and Mann–Whitney pairwise comparisons were conducted to compare the non-parametric data (ARI scores). All statistical analyses were considered significant at a level of *p* < 0.05, except with multiple Mann–Whitney comparisons when performed after the Kruskal–Wallis test, where the Bonferroni correction was applied with a *p*-value ≤ 0.008 in phase I and ≤ 0.025 in phase II results.

## Results

### Testing the new nCaP-SEP formulation at 24 h water storage and post thermocycling

The experimental formulations nHA-SEP and nACP-SEP demonstrated higher pH values of 1.3 and 1.1, respectively, compared to the pH of control SEP (0.8). The SBS outcomes are shown in Table 1, which demonstrates that the control group consistently yielded the highest mean values, followed by lower values in the sequence nHA-SEP > nACP-SEP after both 24 h water storage and 5000 thermal cycles. One-way ANOVA test revealed statistically significant differences (*p* < 0.001) in SBS between the CSEP and the nCaP-SEPs post both ageing methods. Tukey HSD post hoc multiple comparisons demonstrated that incorporating nCaP into the SEP significantly decreased the SBS mean values (*p* < 0.001). There was no significant difference between the two nCaP-SEPs debonded at 24 h water storage, while the nHA-SEP exhibited significantly higher SBS than nACP-SEP post thermocycling. It is noteworthy to mention that nine out of twenty brackets of nACP-SEP group encountered bracket failures during thermocycling, hence only 11 specimens were used to test the SBS.

**Table 1:** Descriptive and inferential statistics of shear bond strengths (SBS) of bracket debonding at 24 h water storage (WS) and post 5000 thermal cycles. Compared with the control group (CSEP), the experimental nCaP groups produced significantly lower mean SBS values following both ageing methods

Ageing method	SEP group	*N*	Mean SBS	SD	Min	Max	Statistics: ANOVA	Statistics: Tukey HSD post hoc multiple comparisons
			(MPa)					
24 h WS	CSEP	20	18.49	4.49	10.73	25.88	*F* = 22.67 *df* = 2 *p* < 0.001	nHA-SEP,nACP-SEP*
	nHA-SEP	20	13.46	2.67	9.76	17.85		CSEP*
	nACP-SEP	20	11.64	2.46	8.73	16.58		CSEP*
Thermocycling	CSEP	20	19.39	5.06	11.52	28.62	*F* = 95.00 *df* = 2 *p* < 0.001	nHA-SEP,nACP-SEP*
	nHA-SEP	20	13.38	2.35	10.3	17.88		CSEP,nACP-SEP*
	nACP-SEP	20 (9BF)	3.12	3.40	0.00	8.96		CSEP,nHA-SEP*

*WS* water storage, *BF* bracket failure.

*Significant differences between groups at the 0.001 level.

**Fig. 2:**
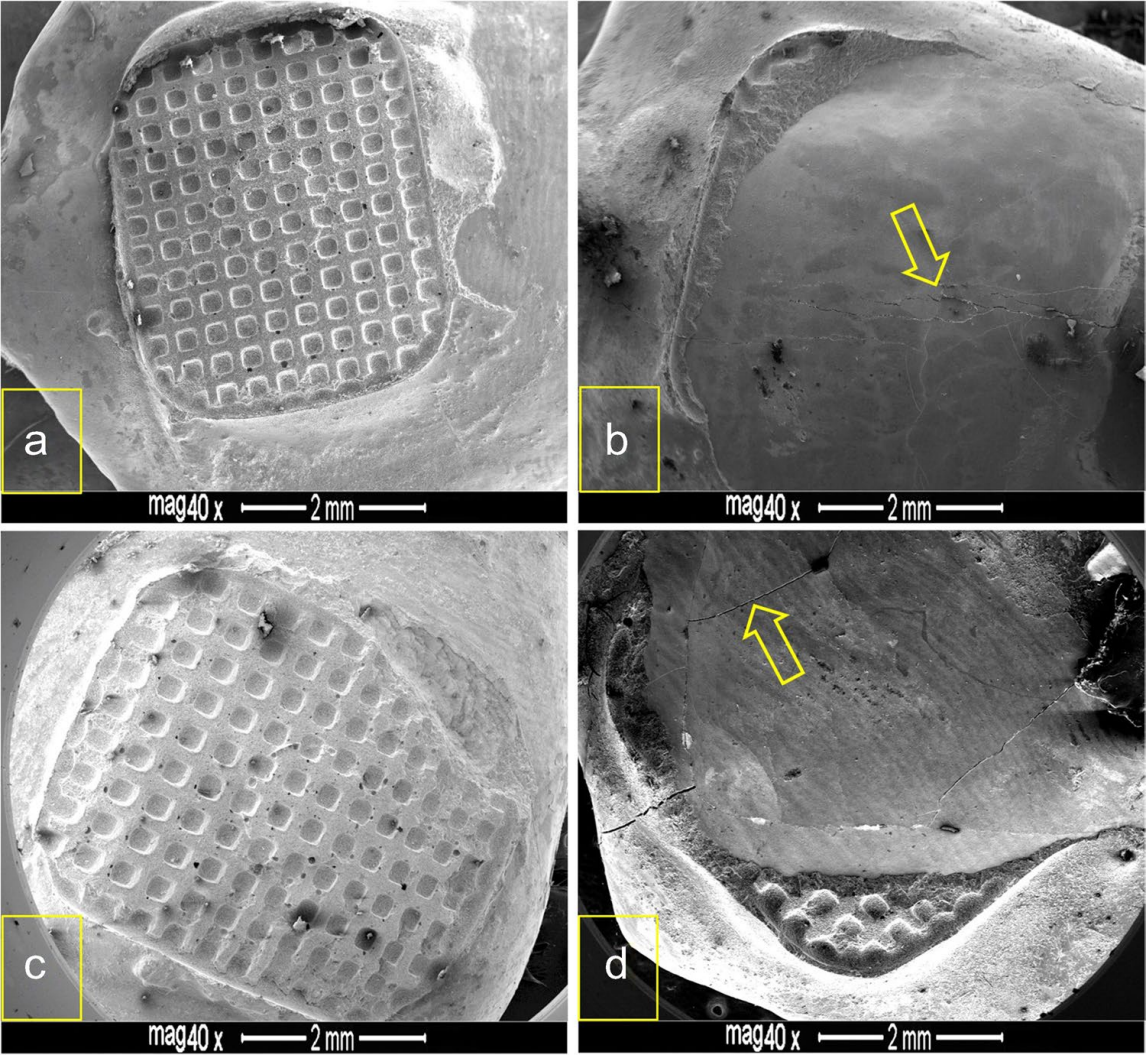
FESEM images of debonded enamel surfaces etched with the control SEP. **a** and **b** Bracket debonding after 24 h water storage. **c** and **d** Bracket debonding post thermocycling. **a** and **c** Retention of all or considerable amounts of adhesive. **b** Chipping of enamel (arrow). **d** Cracking of enamel (arrow) with adhesive retention

**Fig. 3:**
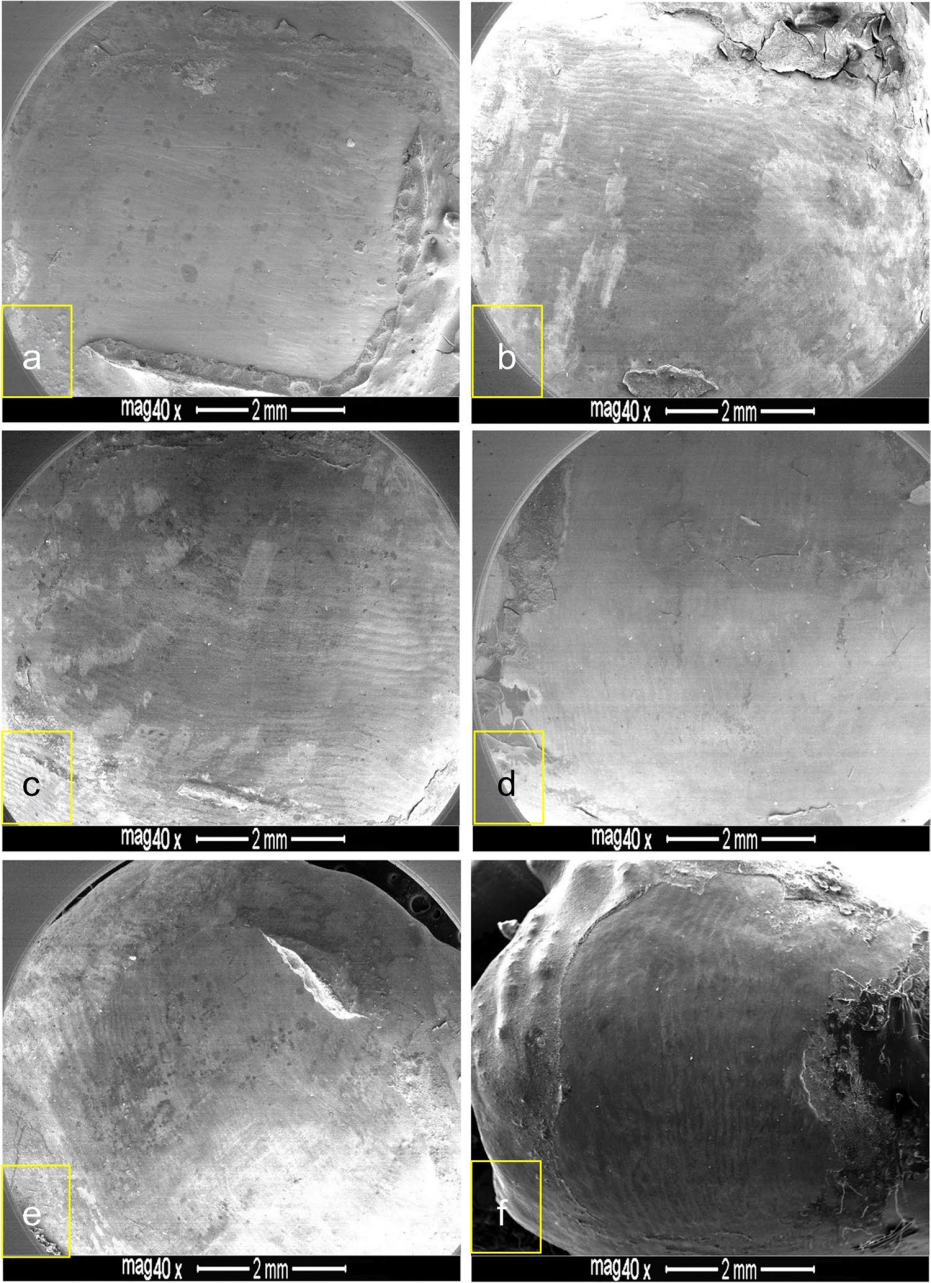
FESEM images of debonded enamel surfaces etched with nCaP-SEP. Immaculate buccal enamel surfaces with no or minimal adhesive retention following etching with 7% nHA-SEP (**a**–**c**) and 7% nACP-SEP (**d**, **f**); bracket debonding was conducted after 24 h storage in water (**a**, **d**, **e**) and post thermocycling (**b**, **c**, **f**)

Upon inspection of the enamel surfaces after brackets debonding, the amounts of adhesive residue in the control group were significantly higher (*p* < 0.001) than in the nCaP-SEP groups as shown in Table 2. The ARI scores in the CSEP group involved both adhesive and cohesive failures with 30% and 35% enamel damage (crack and fracture) at 24 h water storage and after thermocycling respectively, with more adhesive remnants on the tooth surface in thermocycled groups compared to 24 h water storage (Fig. 2). In contrast, the most predominant bond failure for the nCaP-SEP groups was adhesive mode, which remarkably left no or minimal adhesive residue and preserved the integrity of enamel, irrespective of the ageing method (Fig. 3).

**Table 2:** Descriptive and inferential statistics of adhesive remnant index (ARI) scores of bracket debonding at 24 h water storage and post 5000 thermal cycles. The control group (CSEP) exhibited significantly higher scores than the experimental nCaP formulations following both ageing methods

Ageing method	SEP group	*N*	ARI scores				Statistics: Kruskal–Wallis
			0	1	2	3	
24 h WS	CSEP^a^	20	1	14 (3EC + 2EF)	2 (1EC)	3	*H* = 34.74 *df* = 2 *p* < 0.001
	nHA-SEP^b^	20	15	5	0	0	
	nACP-SEP^b^	20	18	2	0	0	
Thermocycling	CSEP^a^	20	2 (2EF)	7 (3EC)	3 (2EF)	8	*H* = 6.6 *df* = 2 *p* < 0.05
	nHA-SEP^b^	20	14	6	0	0	
	nACP-SEP^b^	20	16 (9BF)	4	0	0	

ARI scores: 0, no adhesive left on enamel surface; 1, less than half of adhesive left; 2, more than half of adhesive left; and 3, all adhesive left with a distinct imprint of the bracket mesh. Dissimilar letters (a, b) indicate a statistically significant difference at *p* < 0.025 post-Mann–Whitney multiple comparisons.

*EC* enamel crack, *EF* enamel fracture, *BF* bracket failure, *WS* water storage.

Kruskal–Wallis tests displayed statistically significant differences (*p* < 0.001) in ARI among the three groups post both ageing environments. Mann–Whitney pairwise comparisons demonstrated that the CSEP groups exhibited significantly higher ARI scores than the nCaP-SEP groups debonded at both ageing time points (*p* < 0.025), with non-significant differences between the nCaP-SEP groups.

### XRD

**Fig. 4:**
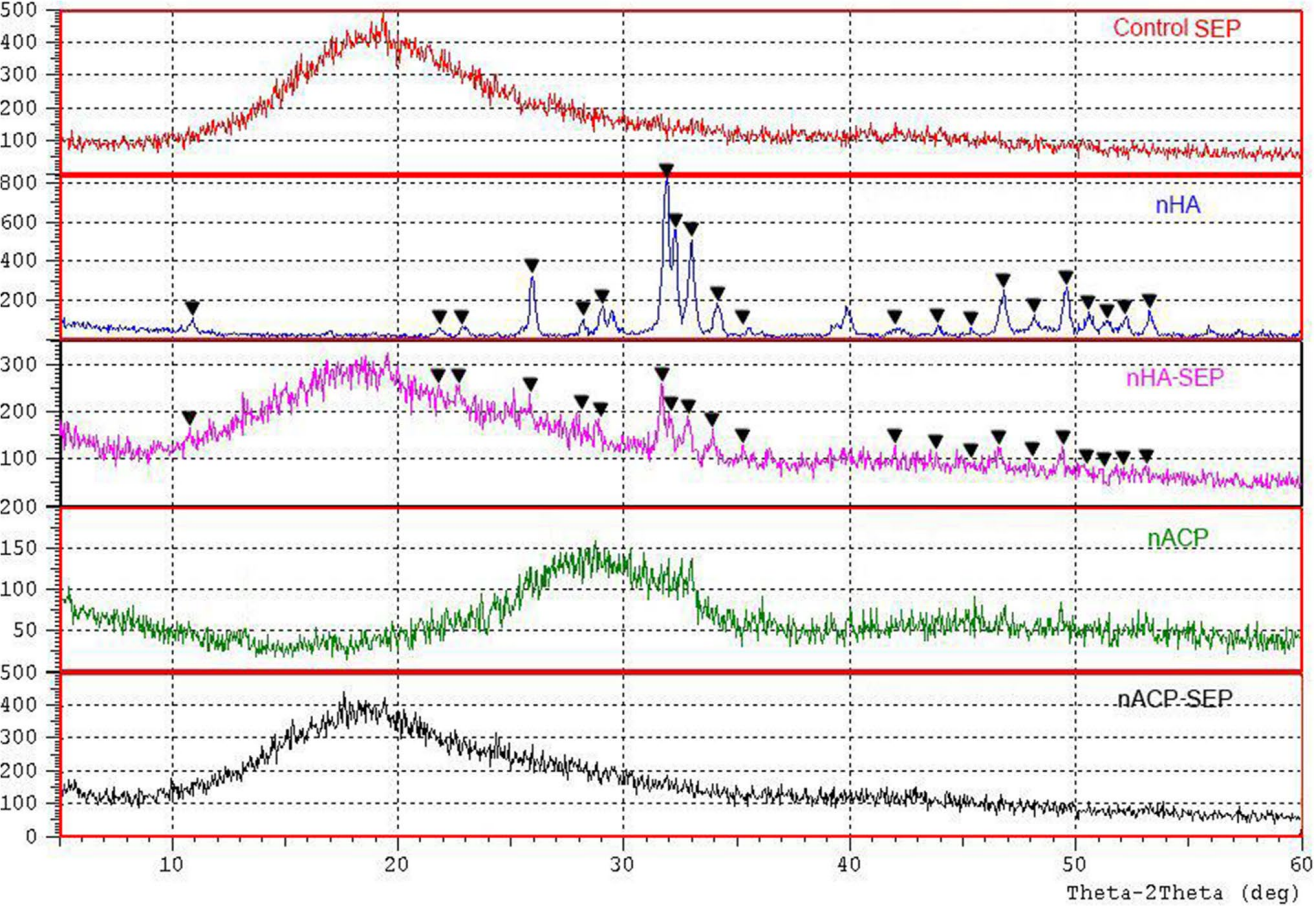
X-ray diffractograms of control SEP, nHA, nHA-SEP, nACP, and nACP-SEP samples. Peaks denoted with triangles are identified as HA single crystalline phase (JCPDS No. 09–0432)

Figure 4 shows XRD patterns of the CSEP resin, nHA, nHA-SEP, nACP, and nACP-SEP. The spectrum analyses yielded identical results for the three samples of each formulation. The XRD pattern of the control SEP (red curve) did not show any sharp diffraction peaks, indicating amorphous non-crystalline properties with non-diagnosed broad XRD diffraction positioned at around 2*θ* = 18–20°. The XRD pattern of nHA (blue curve) and nHA-SEP (pink curve) exhibited different sharp diffraction peaks located at 2*θ* = 10.82 (100), 21.81(200), 22.90 (111), 25.87 (002), 28.126(102), 28.96(210), 31.77(211), 32.19 (112), 32.9(300), 34.04(202), 35.48 (301), 42.02(311), 43.8(113), 45.3 (203), 46.71(222), 48.10 (312), 49.46 (213), 50.49 (321), 51.28 (410), 52.10° (402), and 53.14° (004). These peaks were successfully referenced as a HA crystalline phase with hexagonal symmetry (JCPDS No. 09–0432). The identified diffraction peaks of the HA crystal in the nHA-SEP showed lower intensity than the pure nHA crystals and retained the broad XRD diffraction of the resin. The XRD pattern of nACP (green curve) did not show any sharp diffraction peaks, indicating its amorphous structure. Moreover, the ACP phase was confirmed by the appearance of broad XRD diffraction positioned at 2*θ* =  ~ 30 [19, 32]. However, this identifying band disappeared when the nACP powder was incorporated into the SEP resin (black curve) while retaining the resin’s broad XRD diffraction.

### FESEM examination of nCaP-SEP

**Fig. 5:**
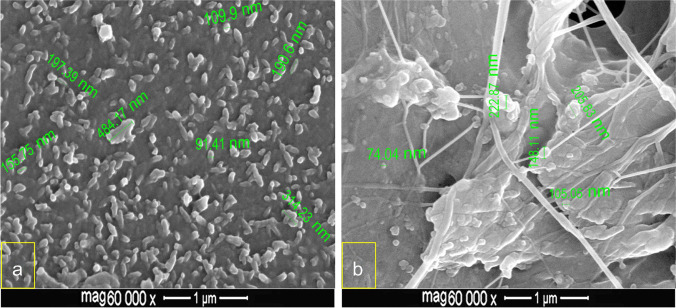
FESEM images displaying the distribution of nanoparticles within the polymerized experimental SEPs. **a** nHA-SEP: spherical or rod-shape nHA are regularly distributed in isolated and agglomerated forms of particle size below 200 nm, and agglomeration size up to 484.17 nm; **b** nACP-SEP: spherical nACP showing more dispersion with isolated particle size below 150 nm, and agglomerated particles up to 222.87 nm

The morphology and distributions of nHA and nACP in the experimental SEPs are presented in Fig. 5. The images revealed dispersion of the nanoparticles within both synthesized resin matrices; however, the nHA showed more regular and condensed arrangements. Both types of nanoparticles were presented in isolated and agglomerated forms. The isolated nHA exhibited nano-sphere or nano-rod shapes of a crystal size less than 200 nm, while the size of crystal agglomeration reached up to 484.17 nm. On the other hand, the isolated nACP particle elicited spherical shapes of a size below 150 nm, while the crystal agglomeration recorded up to 222.87 nm.

### FESEM examination of etch-patterns post conditioning of the enamel

**Fig. 6:**
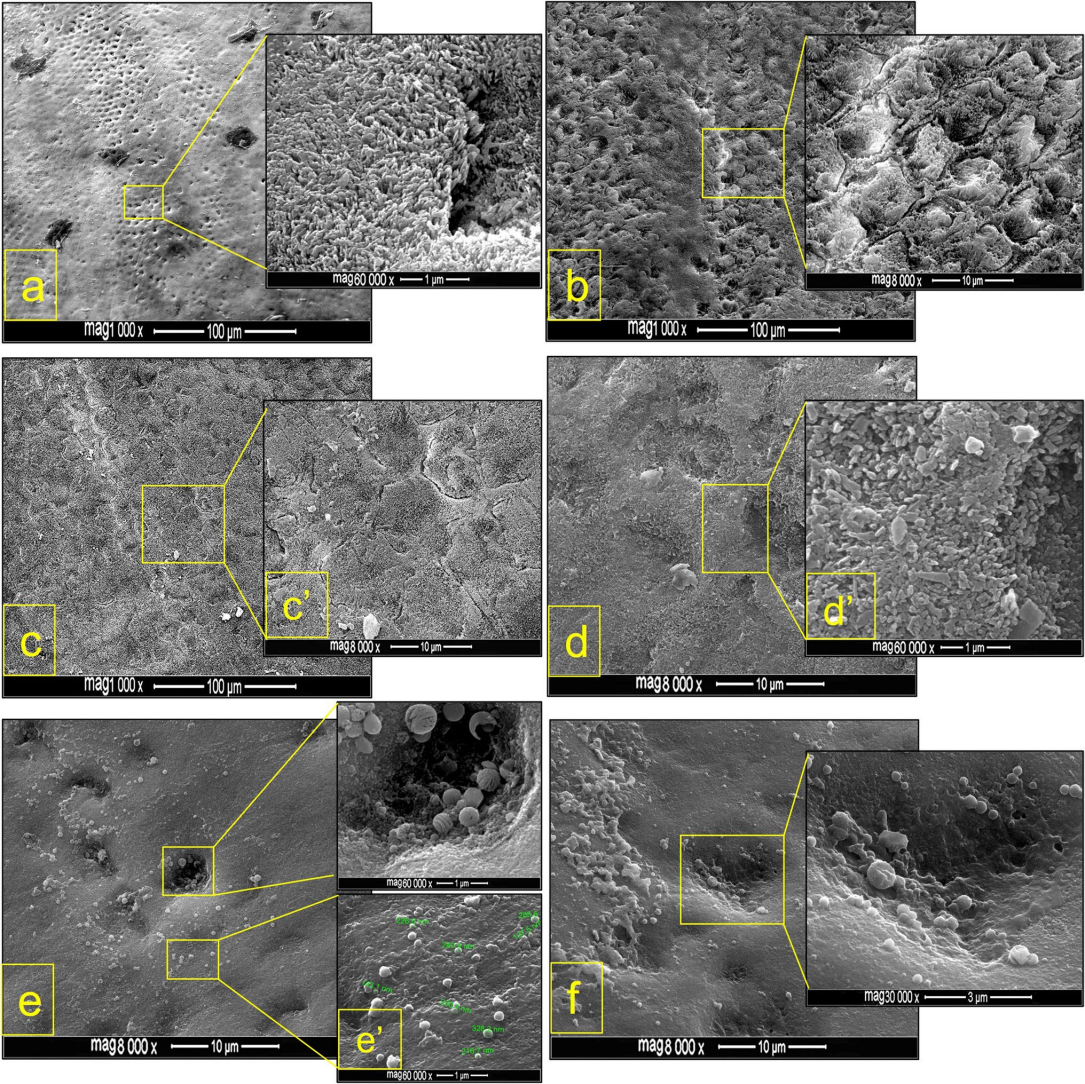
FESEM images showing the etch-patterns of premolar buccal enamel surfaces etched with the control (CSEP) (**a**, **b**), 7%nACP-SEP (**c**, **d**), or 7%nHA-SEP (**e**, **f**). Etching with CSEP yielded open micro-pores with etch-patterns varied from type 2 (**a**) to type 3 (**b**); etching with 7%nACP-SEP displayed mostly type 3 etch-pattern (**c**, **d**) with nCaP precipitates (**c′**, **d′**), while the 7%nHA-SEP revealed a milder etching effect presenting smooth surfaces (**e**, **f**) with evident nCaP precipitations in the form of small granules (**e′**) or larger aggregations (**f**), with partial or complete micro-pores obliteration (**e**, **f**)

The resultant enamel etch-patterns were analyzed by the etch-pattern scale. Enamel conditioning with the CSEP yielded roughened surfaces with well-defined open micro-pores and a predominant cobblestone pattern, which is the ideal type 2 etch-pattern (Fig. 6a), in addition to type 3 etch-pattern, which is a mixture of the honeycomb (type 1) and cobblestone (type 2) appearance (Fig. 6b). Etching with nACP-SEP exhibited almost similar etch-patterns to the CSEP with scattered small sized mineral deposits (Fig. 6c, d). In contrast, the nHA-SEP resulted in smoother surfaces depicting a combination of types 4 and 5 etch-patterns with evidence of nCaP precipitates. The nCaP particles were seen as small granules in a range of 137.5–328.2 nm (Fig. 6e) or a mixture of clustered precipitations featuring as grains or lamellar-like aggregations (Fig. 6f). Moreover, on a larger scale inspection (magnification × 30,000), the images interestingly showed the ability of nCaP to fill the enamel micro-pores eliciting zones of mineralized focal holes (Fig. 6e,f).

### Assessing the re-mineralization potential of the new etchant system using RS

**Fig. 7:**
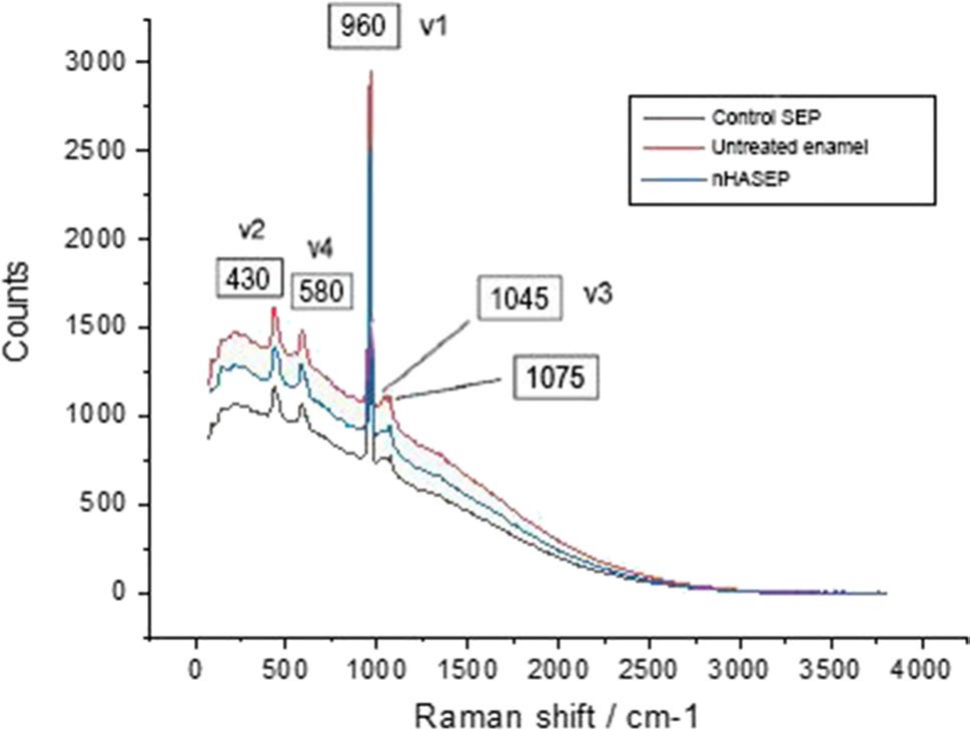
Raman spectra of untreated enamel (red curve), enamel treated with 7%nHA-SEP (green curve), and enamel treated with the control SEP (blue curve). The spectra of the three areas depict five characteristic bands: four phosphate ion vibration modes (ʋ1-PO at 960 cm^−1^, ʋ2-PO at 430 cm^−1^, ʋ3-PO at 1045 cm^−1^, and ʋ 4-PO at 580 cm^−1^), and B-type carbonate ion at 1075 cm^−1^. Raman peak at 960 cm^−1^ typifies tetrahedral PO4 group (P–O bond) as the highest phosphate peak within HA; the untreated enamel zone demonstrated the highest intensities at all phosphate peaks, followed by nHA-SEP and CSEP consecutively, signifying the dissolution of HA during enamel treatment

Raman mapping (3 × 4 matrix) was conducted over the central part of each of the three enamel zones of the ten flat buccal molar surfaces. All examined enamel surfaces exhibited the five distinctive peaks of hydroxyapatite (HA): one peak for the B-type carbonate ion (CO_3_^2−^) at ~ 1075 cm^−1^, and the other four peaks represented the internal vibrational modes of the PO_4_^3−^ ion including the symmetric stretching mode (*v*_1_ at ~ 960 cm^−1^), doubly degenerate bending modes (*v*_2_ at ~ 430 cm^−1^), triply degenerate asymmetric stretching modes (*v*_3_ at ~ 1045 cm^−1^), and triply degenerate bending modes (*v*_4_ at ~ 580 cm^−1^) (Fig. 7). The strongest peak in untreated and conditioned enamel spectra was that of *ν*_1_ at ~ 960 cm^−1^, yet the intensities of the four PO_4_^3−^ peaks were different between the untreated and conditioned zones. The untreated enamel surfaces exhibited the highest mean values of the four PO_4_^3−^ peak intensities, and each was found to decline post conditioning with the SEP in the order CSEP < nHA-SEP (Table 3). Depending on the peak intensity mean values, enamel etching with nHA-SEP yielded less demineralization in comparison with the CSEP, denoting a potential remineralization effect. However, one-way ANOVA revealed statistically non-significant differences in the four PO_4_^3−^ peak intensities among the three zones (*p* > 0.05), as shown in Table 3.

**Table 3:** Intensities of four phosphate Raman peaks of a flat enamel surface divided into three zones: untreated enamel versus treatment with two SEPs, displaying the highest intensity means in the untreated enamel zone, yet statistically non-significant differences

Zones	Peak symbol	Peak position (cm^−1^)	No. of samples	Mean of intensity	SD	Min.-max
Untreated enamel (a)	V2	431	10	1814.0	643.3	1078.1–2856.8
	V4	591	10	1649.1	697.3	963.2–2971.5
	V1	961	10	2757.1	671.6	2029.9–4213.6
	V3	1045	10	1209.5	446.2	817–2028
CSEP (a)	V2	431	10	1371.6	572.0	598.2–2281.4
	V4	591	10	1273.8	535.9	551.2–2089.8
	V1	961	10	2203.0	578.3	1228–2959.5
	V3	1045	10	928.8	393.6	351.1–1447.3
nHA-SEP (a)	V2	431	10	1691.3	596.7	948.7–2902.7
	V4	591	10	1567.3	603.3	914.5–2774.2
	V1	961	10	2657.9	551.9	1668.1–3637.2
	V3	1045	10	1158.9	392.0	729.8–2052.6

- Non-significant differences (*p* > 0.05) in the mean intensities of four phosphate peaks post one-way ANOVA test.

## Discussion

Enamel conditioning with SEPs has recently attracted ample interest in orthodontic bonding, as they can etch and prime the enamel surface in a single step; this addresses the increased demand for safer, more convenient, and less technique-sensitive materials, but has not eliminated the enamel damage occurring at bracket debonding [16, 33]. The hypotheses of the current study were proven and accepted, since a novel bioactive, enamel-preserving orthodontic SEP left minimal or no adhesive residue with a potential remineralizing effect on the enamel and did not compromise orthodontic bond strengths.

The mechanism of the SEP performance can be explained with adhesion–decalcification concepts and comprises two phases. When the SEPs come first in contact with the enamel, the acid (functional monomer) bonds ionically to calcium (Ca^2+^) of the HA in phase 1; this is accompanied by the release of PO_4_^3–^ and hydroxide ions from the HA into the SEP solution. Two options are possible in phase 2 after this ionic bonding step in phase 1: either the bond is hydrolytically stable and produces a calcium-monomer salt that can copolymerize with the monomers of the adhesive resin, or if the chemical reaction is unstable, decalcification and release of (Ca^2+^) and (PO_4_^3−^) ions from the enamel surface take place. The latter option occurs with strong SEPs (of pH ≤ 1) and results in filling of micropores with the flowing resins by capillary action; this yields micromechanical locking, a condition that is similar to that of the CAE technique [34, 35]. By adopting the latter scenario, the rationale of the current study was premised on incorporating nHA and nACP into the SEP monomer to create a SEP solution rich in (Ca^2+^) and (PO_4_^3−^) ions and capable of terminating phase 2 rapidly, thus hindering further depletion of (Ca^2+^) and (PO_4_^3−^) ions from the enamel surface.

The optimal strength for bonded brackets is expected to survive bracket failure throughout treatment while preserving the enamel integrity at bracket debonding [15]. Although there are no unanimously accepted values for optimal orthodontic bonding strength, a minimum range of 5.9–7.8 MPa was deemed adequate for bracket bonding while attaining satisfactory clinical performance [36]. A higher range up to 20 MPa was suggested [37], yet it has been reported that a SBS higher than 12 MPa can induce enamel damage [15, 16]. Thus, a range of 6–12 MPa has been adopted in the majority of in vitro orthodontic bond strength studies [38, 39]. In this study, the SBS values of the developed nHA-SEP were within this range.

Seven percent weight fractions were selected for both nCaP types based on a preliminary range-finding phase, in which three different percentages of the two nCaP compounds (5%, 7%, and 9% wt) were adopted for the weight:weight ratios and added to the plain SEP. Assessments of formulation performance were used to determine the best results for the SBS with the least enamel damage and AR on enamel surface. Adding nCaP powders to the SEP decreased the SBS significantly and imposed a buffering effect by increasing the pH of the strong plain SEP (pH = 0.8) in both nCaP-SEP formulations. This buffering alleviated the etching effect of the developed SEP. A comparable effect was found in a recent study when CaP powder was added to a phosphoric acid solution [11]. In addition, the irregular distribution or agglomeration of these nanoparticles might form defect points that interrupt resin curing and generate sites for crack propagation within the material, hence adversely affecting its mechanical properties [22]. Moreover, nanofiller clusters might increase the viscosity, impede the flow of the resin into the micropores and yield lower bond strengths [40].

On the other hand, the nanoparticles added into the SEP could protect the enamel structure at bracket debonding. The acidic medium of the plain SEP dissolves the added nCaP, which in turn effectively fills the micropores created on the enamel surface by discharging inorganic ions [17, 41]. The released Ca^2+^ and PO_4_^3–^ ions move into the interprismatic spaces and are converted into crystals of HA during the demineralizing-remineralization process [41]. Stagnation of these nCaP crystals at high pH could resist hydrolytic degradation due to the low solubility and the bulk of crystallites [27]. A similar effect was demonstrated in recent work, in which stagnation of CaP crystals at micropore openings obstructed ingress of the resin into the micropores, hence intentionally decreasing the bond strength to preserve the enamel integrity [11]. It is logical to assume that the reduced SBS associated with the nHA-SEP compared to the control SEP would not threaten the underlying bonded enamel, as the impeded resin penetration would be replaced by precipitated nCaP rather than leaving the micropores empty and open to acidic and hydrolytic attack. In addition, the added nCaP can react competitively with the functional monomer, hence hindering aggressive demineralization by the plain SEP. These outcomes were eventually confirmed by FESEM and Raman analyses.

Enamel conditioning with the control SEP revealed significantly higher ARI scores than the nHA-SEP group post bracket debonding, with signs of enamel damage (seven samples showed cracked enamel and six exhibited enamel chipping). In contrast, etching with nHA-SEPs left immaculate enamel surfaces with little or no adhesive retention. After both aging protocols (24 h and 5000 thermal cycles), the overall ARI scores indicated weaker enamel-adhesive interface postetching with nHA-SEP than with the control SEP, whereas both SEP groups demonstrated similar increased overall ARI scores after thermocycling, which may be attributed to an increase in the adhesion of both primers due to water sorption-induced polymer expansion [42]. The chemistry of the plain SEP was manipulated by incorporation of the nCaP to weaken the enamel-adhesive interface, which shifted the bond failure site to the enamel surface without jeopardizing its integrity, hence enhancing the potential for reduced chair-side time and cost at the post debonding clean-up stage.

Quantitative assessments of enamel mineral content were performed with Raman microscopy since the signal intensity is proportional to the number of molecules within the volume of the scanned area [31]. The Raman spectral observations in the current study exhibited the five distinctive peaks of enamel in all sound and conditioned surfaces, in accordance with previous studies [11, 19]. However, the conditioned enamel zones yielded lower intensities for PO_4_^3−^ peaks than the unconditioned zones, and the PO_4_^3−^ intensities were higher for zones treated with nHA-SEP than with CSEP, confirming nCaP reprecipitation (demonstrated by FESEM) after enamel treatment with the nHA-SEP; these outcomes can be explained by etching aggressiveness. It has been reported that significant depletion of the intensity for the PO_4_^3−^ peak can occur with surfaces etched with CAE or in the area corresponding to the body of an enamel lesion [11]. In contrast, the intensity of the PO_4_^3−^ peak at the end of an incipient lesion converges to that of intact enamel [31, 43]. Since SEPs exhibit a relatively milder etching pattern than carious lesions and CAEs [16, 44], the SEPS preclude significant depletion of the mineral content.

Resistance of an orthodontic bonding system to degradation is essential for the success of orthodontic therapy. Addition of a small amount of nanofiller might enhance SEP longevity, as inorganic nanoparticles are less liable to degradation than the polymeric matrix [45]. Modifying nanofiller size, shape, composition, or amount can improve resin mechanical properties [40]. The resistance of CSEP and nCaP-SEP to degradation was tested by thermocycling [11, 46]. The addition of nCaP to the plain SEP significantly reduced the SBS values, yet the nHA-SEP showed acceptable SBS values at both 24 h and after thermal stresses were applied. The nACP-SEP group, however, did not survive the thermal cycling protocol. Hydrolytic degradation caused by ample and rapid thermal changes stressed the tooth-bracket joints at the enamel-adhesive interfaces (ARI score 0) and led to unacceptably low SBS values. The latter finding was unexpected, as the nACP-SEP had a lower pH (1.1) than nHA-SEP (1.3) and demonstrated a pronounced and more aggressive etching pattern. In addition, the FESEM analysis showed less agglomeration and scattered distribution of nACP, both within the resin and on the enamel surface, after etching. The difference in the performance of nHA-SEP and nACP-SEP might be attributed to differences in their structural properties and binding affinities with the enamel. It has been reported that nCaP with a high degree of crystallinity showed improved mechanical properties; hence, adding nHA to an adhesive may enhance its properties [47], since HA is the most stable and least soluble form of the CaP family [48]. In addition, nHA has a structure similar to those of enamel primary building blocks, so there is a pathway for biomimetic binding [18]. They have a relatively high interfacial energy that enables better adsorption to enamel and resists detachment. In contrast, the lower adsorption of nACP by the enamel may be explained by the dissimilar structures and chemical compositions of nACP and the enamel apatite [49].

Phase identification for HA crystals in the developed nHA-SEP was done with XRD analysis; the diffraction intensities observed were lower than those of the pure nHA sample, which might simply reflect the different nHA weight fractions of the different samples. On the other hand, the XRD analysis of nACP-SEP showed no crystal phase transformations of nACP after incorporation into the plain SEP. This finding might be attributable to the pH of the nACP-SEP primer, as ACP phase transformation into a more stable apatite crystalline form was sensitive to slight drops in pH from 7.6 to 6.8, both of which are far above the pH of nACP-SEP (1.1) [32, 50]. The change in pH implies the operation of a chemical reaction within the incorporated primers [32]. However, the XRD pattern did not show any CaP phase after transformation into nACP-SEP, which might be related to a low percentage for which the band was overwhelmed by the amorphous band of the CSEP or to other structural transformations.

An ideal scenario for confirming bonding system efficacy is an accurate imitation of the oral environment, although this is an extremely challenging goal due to variables related to the patient and the challenges of clinically distinguishing the numerous factors affecting the performance of a particular bonding system [38, 39]. In addition, in vitro studies allow standardized procedures that can test a particular bonding system and are worthwhile for at least primary evaluation and selection of materials. This study involved in vitro screening of a developed orthodontic bonding system prior to further verification with a controlled in vivo investigation, and the outcomes obtained after laborious testing show promise for successful clinical performance.

## Conclusion

A novel bioactive orthodontic self-etch system capable of producing clinically acceptable bond strengths was developed, which adopts a three-in-one EPR (etch-prime-remineralize) approach. In contrast to the traditional paradigm of implementing remineralizing nanoparticles into the body of adhesives to release Ca^2+^ and PO_4_^3–^ ions, which incompletely apprehend the clinical concerns with a non-significant control on enamel damage, the new approach incorporates nHA particles into a self-etchant to precipitate directly on the enamel; the target area for preventive attempts. The unprecedented combined advantages of this new bioactive SEP offer a minimally invasive bonding system that is expected to preserve the enamel with clinically acceptable bond strengths.
